# Optimization of competitive supply chains with retailers’ horizontal cooperation and consumers’ green preference

**DOI:** 10.1007/s11356-021-14192-y

**Published:** 2021-07-16

**Authors:** Wenfang Shang, Liangliang Teng, Jian-bo Yang

**Affiliations:** 1 School of Business, Zhengzhou University, Zhengzhou, China; 2 AMBS, University of Manchester, Manchester, UK

**Keywords:** Cooperation between retailers, Consumers’ green preference, Competition intensity, Competitive supply chains

## Abstract

With the wider recognition of the concept of environmental protection and sustainable development, more and more manufacturers have begun to implement green manufacturing strategies. However, green development is a gradual process, and the coexistence of ordinary and green products is common. This paper examines the competition between ordinary products and green ones based on supply chains, and discusses the impact of retailers’ horizontal cooperation on pricing, greenness, market demand, profit, and other related decisions and results. Model solutions and numerical experiments have shown that consumers’ green preference (CGP) has a positive impact on the results of green products and negative effects on those of ordinary ones; however, the impact of competition intensity (CI) is more complex, and the trend tends to change if it exceeds a certain critical value. In general, cooperation encourages retailers to raise prices and make manufacturers lower wholesale prices, but the retail and wholesale prices of green products are always higher than the corresponding prices of ordinary ones. Market demand can be reduced due to cooperation, but it is conducive to strengthening green products’ greenness. However, if CI exceeds a certain threshold, the greenness will be weakened by cooperation. Both retailers can benefit from cooperation easily and they always reach a win-win situation, but manufacturers suffer badly as a result. Nevertheless, when CI is weak, cooperation will bring a greater profit improvement to the whole chain of ordinary products; in this case, if a retailer is willing to compensate for a manufacturer’s profit loss and help it benefit from cooperation, the manufacturer will encourage the retailer to cooperate horizontally with the green retailer, but the green chain will be seriously damaged. When CI is strong, the greenness of green products is not obvious enough, and cooperation can bring some chances to achieve a win-win situation for two chains; if the green retailer can obtain a larger share from cooperation under this scenario, it can also enable its manufacturer to obtain compensation and change the profit increment from negative to positive. From an overall perspective, when CI is very weak or very strong, horizontal cooperation is conducive to improving the profit of the entire supply chain competition system, but when CI is relatively flat, independent operation for each chain is more advantageous; if the competition status is stable and unchanged, independent competition will be superior to horizontal cooperation.

**Supplementary Information:**

The online version contains supplementary material available at 10.1007/s11356-021-14192-y.

## Introduction

In order to seek sustainable development, manufacturers have to consider green production and operation, such as GE, Lenovo, and Linglong Tire (Yang et al. 2020), which extend to green supply chain management. Green supply chain (GSC) has attracted widespread attention from academic field and management practice, which mainly focus on how to make decisions on product greenness (Ghosh and Shah 2015; Li and Li 2016; Zhu and He 2017; Moradinasab et al. 2018; Nakandala and Lau 2019; Jian et al. 2019; Murali et al. 2019), how to coordinate GSCs (Ghosh and Shah 2015; Du et al. 2015; Yang et al. 2017; Hong and Guo 2019; Yang and Gong 2021), how CGP impacts on green decisions (Pu et al. 2018; Hong et al. 2018; Zhang et al. 2020; Adhikari and Bisi 2020; Ma et al. 2020; Liu et al. 2012), etc. One of the unavoidable problems in the development of GSC is how to compete with ordinary supply chain (OSC) with non-green products. The improvement of material living standards has pushed consumers to choose green products with stronger quality attributes, and the increase in environmental awareness has also made a considerable proportion of consumers willing to spend more on buying green products. Therefore, ordinary product manufacturers naturally find ways to improve the green attributes of their products, such as introducing environmentally friendly raw materials^1^, launching green production processes^2^, and signing green channel agreements with retailers^3^. But we understand well that this requires a huge expense (Yalabik and Fairchild 2011) and a long cycle, which cannot be accomplished overnight^4^. Thus, manufacturers of ordinary products will still exist and compete with those of green ones for a few months even a few years, based on their respective supply chains. For instance, both energy-saving and ordinary appliances have penetrated into thousands of households, both green food and ordinary food have their own market share, and both new energy vehicles and petrol ones are running smoothly on the road. Many researches have paid attention to the competition and substitution between green and non-green products in their research, such as Zhang et al. (2014), Yenipazarli and Vakharia (2015), Hafezalkotob (2015), Basiri and Heydari (2017), Madani and Rasti-Barzoki (2017), Hafezalkotob (2017), Yang et al. (2017), Pu et al. (2018), Jamali and Rasti-Barzoki (2018), Meng et al. (2021), etc. Naturally, a new question arises: Can green and ordinary products be sold by the same retailer?

First of all, as far as home appliances are concerned, green products^5^ are those that consume less energy, save water and electricity, reduce noise, and emit less pollution. Our visits to retail stores show that retailers like Yongle Appliances display washing machines of XIAOYA, YONGLE, Royalstar, Whirlpool, DIQUA, Panasonic, Media, SIEMENS, and other brands in the same exhibition hall; the first three brands have energy efficiency labels of Level 4, Level 3, and level 2, respectively, while the latter five ones all have labels of level 1. With respect to refrigerators, SHANGLING, Haier, Media, and other brands are also displayed in the same zone, while the energy efficiency label of SHANGLING is level 2 and that of the latter two is level 1. Focusing on Gome Online Mall, Suning.com, and JD.com, we also found that multiple brands with different energy efficiency labels coexist. For wall-hung boilers, Chinese market is dominated by Italian brands such as Beretta and domestic brands such as Media and Haier, of which the energy efficiency levels are different, but a retail store usually only sells one brand. Secondly, in terms of food, taking fruits and vegetables for example, green products refer to those are pollution-free and of high quality, which can be certified to use green trademarks. We found that retail stalls in the farmer’s market often coexist with green and ordinary products; in large fresh supermarkets like Yonghui, fruits and vegetables are often sourced directly with strict process from greenhouse base, where product quality and safety can be guaranteed, and all agri-products are green ones. Retailers like Baiguoyuan have always abided by the “Four Degrees-Taste-Safety” standard and only sell green brand products. Fruits and vegetables, displayed on Dennis Department Store’s underground floor, are all green ones. Thirdly, as for automobiles, NIO new energy vehicles and NIO Day (Customer Experience Center) are popular in China. The current NIO Day cannot go well without a third-party retailer such as traditional 4S stores, which usually coexist with non-green vehicles and other brands. That is, NIO and a third-party retailer cooperate to sell both NIO new energy vehicles and non-green vehicles of other brands. However, NIO Company is committed to replacing third-party retailers and operating NIO Day independently because NIO Day only targets customers who choose NIO new energy vehicles, allowing them to enjoy a variety of home-like experiences. This shows that two different retailers first cooperate and then will work independently.

The above examples tell us that some retailers sell green and ordinary products in the same store, but some ones only sell green products, and some retailers originally cooperate but then split into two parts. A question is why some retailers sell green and non-green products in the same store, but some just sell green ones. A further question is which way is better for the entire supply chain and its two members? To address these questions, we will examine two independent supply chains, with each chain having its own manufacturer and retailer, which are in competition because they provide ordinary and green products respectively. The original problem is reduced to whether two retailers from different chains will cooperate horizontally. Cooperation here involves merge or channel combination. Besides, many retailers may have not only physical stores but online ones as well. They sell products online with different quality from those in physical stores. Products can be supplied by different types of manufacturers such as green and ordinary ones. Online and physical stores are often accounted for independently and considered different retailers. Cooperation between these two types of stores is inevitable, which is common not only in manufacturing supply chains but also in service supply chains. For instance, Wang and Liu (2019) and Alvarez-SanJaime et al. (2013) found that carrier companies not only negotiate with upstream ports in the same supply chain but also interact horizontally with carriers in other chains.

Thus, it is necessary and interesting to gain a more thorough understanding of how competition and cooperation between two chains affect price strategies, greenness, and profitability. People may ask that how to describe competition and cooperation. Products in two competing chains, to some degree, often can be substituted by each other. The higher the substitutability, the fiercer the CI between two chains is. In this paper, we use product substitutability to denote CI. Here we refer to McGuire and Staelin (1983) to introduce competition into market demand functions. Besides, Yu et al. (2016) reported that 75% of Europeans, in 2008, would buy green products, up from 31% in 2005, which means consumers have green preference. CGP in the paper is denoted as sensitiveness to green products and appears with greenness in demand function of green products. Our empirical study shows that although consumers are still price sensitive, but if they fully understand energy-saving and emission-reduction indicators of different products, they tend to choose green ones. For example, it is easy to impress consumers by using indicators such as energy saving and noise of air-conditioning, preservation time and power consumption of refrigerators, gas utilization rate, and exhaust temperature of water heaters. This shows that consumers’ sensitivity to green products will increase the market demand for green products but inhibit that for ordinary ones. Therefore, we refer to Li and Li (2016), Jamali and Rasti-Barzoki (2018), and Yang et al. (2020) to introduce consumers’ sensitivity to green products not only into the demand function of green products but into that of ordinary ones.

Based on the above statement, this paper will investigate whether cooperation between retailers can help raise or reduce the demand for green products and benefit or harm the chains easily. Cooperation here involves that two retailers make price decisions together, not independently. There may be someone considering cooperation as combination. To some degree, the paper will investigate whether it is necessary to combine two retailers into one and why only tens but not all of Gome’s retailers are permitted to sell green home appliances. Thus, comparisons of decisions between cooperation and non-cooperation are necessary.

Specifically, this paper will focus on examining how CGP and CI influence the corresponding strategies of each chain before and after cooperation. The following questions will be addressed:

- *How do CGP and CI influence wholesale and retail prices? What are their differences between cooperation and non-cooperation? What about the greenness of green products before and after cooperation?*
- *What about market demands in two chains? Will they be harmed after cooperation?*
- *What conditions can make both retailers be willing to cooperate? Would CI and CGP influence retailers’ cooperation willingness?*
- *Can manufacturers benefit from retailers’ cooperation?*

There is usually a fixed mindset that cooperation is a good way to improve both partners’ performance. Is it working for two competing systems? When two supply chains compete with each other but retailers determine to cooperate, the following questions naturally arise:

- *What about each whole chain’s profit under cooperation?*
- *Is there a possibility that one chain performs better than the other under cooperation?*

Based on demand functions for each chain and profit maximization with Stackelberg game, theorems, propositions, and numerical results will be given to illustrate the trends of prices, greenness, demands, and profits. How cooperation can help retailers obtain Pareto improvement and how it impacts market demands and manufacturers’ profits will be demonstrated as well.

The rest of the paper is arranged as follows. “Literature review” reviews the related literature from two aspects. Problem descriptions and assumptions are given in “Problem descriptions and assumptions.” Mathematical models and results under cooperation and non-cooperation follow in “Models and solutions,” and optimal decisions generated before and after cooperation will be given and analyzed in tables, theorems, and propositions. “Numerical analysis” gives considerable and detailed numerical examples and the corresponding analysis. Conclusions are made in “Conclusions and Policy Implications.” Appendix 1 lists some notes used in models and analysis of the paper. Proofs of theorems and propositions are enclosed in Appendix 2.

## Literature review

This paper addresses the problems on GSC management. CGP involves with consumers’ environmentally friendly behavior; competition and cooperation involve with literature about competing supply chains. Thus, relevant literature is reviewed from two aspects as follows.

### Supply chain management with regard to consumers’ environmentally friendly behavior

In the areas of customers’ environmental awareness, there are a considerable number of studies. For instance, Biswas and Roy (2015) examined the impact of consumer behavior on emerging economies by exploratory study with green products; Zhang et al. (2020) observed that both firms prefer to invest in emission abatement under a sole channel structure if consumers have eco-friendly preference. Liu et al. (2021) proved that consumer preference for low-carbon products can benefit both supply chain enterprises while achieving the aim of carbon emission reduction. However, Damigos et al. (2020) stated that green fridges are not so easy even hard to sell and there are some other factors influencing demand apart from CGP, such as currency tag and heterogeneity of consumer behavior. The studies tell that environmental awareness is closely related with consumers’ purchasing behavior but they do not pay attention to retailers’ behavior when two different types of products coexist and compete. This paper will consider retailers’ cooperation behavior and examine how it affects demands and pricing strategies of both chains.

Many researchers have treated greenness of environmental friendly products as a decision variable, or examined how consumers’ environmental awareness influences on optimal decisions. Du et al. (2015) studied the impact of emission “cap-and-trade” mechanism with the emission permit supplier and the emission dependent firm. Swami and Shah (2013) assumed that if both supply chain members make efforts to green their operations, the ratio of the optimal greening efforts for the manufacturers and retailers will be dependent on the ratio of their green sensitivity ratios over green cost ratios. Zhang et al. (2015) studied the impact of consumer environmental awareness on decisions and channel coordination. Ghosh and Shah (2015) examined coordination issues and the impact of cost-sharing contracts on key decision variables. Chen et al. (2017) investigated centralized decisions on pricing and production for green crowd funding products with different quality. Basiri and Heydari (2017) investigated the green sales effort and green quality decisions of the retailer and manufacturer, respectively. This study also considers the effects of consumer environmental awareness in green product pricing. Pu et al. (2018) uncovered the influence of CGP on price and demand and the relationship between the influence coefficient of retailers’ promotional effort on consumers’ utility and retailer profits. Hong et al. (2018) investigated a green-product pricing problem by taking into account consumer environmental awareness and ordinary product reference. The green product’s pricing strategy is significantly affected by asymmetric information. Chen et al. (2019) studied the reverse logistics pricing strategy of GSC. Zhang et al. (2020) demonstrated that consumer’s eco-friendly preference influences both manufacturer and retailer’s choice of selling low-carbon products or not. Adhikari and Bisi (2020) considered green quality as decision variable to discuss centralized and decentralized settings. Ma et al. (2020) considered Stackelberg pricing game and simultaneous game to illustrate how carbon emission constraint affects GSC’s operation strategy. The researches give some insights but are not about two separate competing supply chains with consumers’ environmental awareness. There are also some studies on competition between independent chains (Zhu and He 2017; Jamali and Rasti-Barzoki 2018; Yang et al. 2020; Meng et al. 2021), but they believed that the impact of greenness on green demand is always greater than that on ordinary ones.

Based on the researches above, we will denote consumers’ environmental awareness as CGP and focus on how to make optimal decisions with CGP, which has the same impact on both ordinary and green products’ demand.

### Cooperation and competition between different supply chains

Most of the literature in this area is about how to cooperate with internal members in competing supply chains; however, there is also a stream especially considering retailers’ external competition and cooperation. David and Adida (2015) examined a supply chain’s competition and coordination in which a single supplier has multiple different retailers. Huang et al. (2016) studied the price competition and cooperation policies when one manufacturer has two duopoly retailers. Chen and Xiao (2017) also considered retailers’ cooperation or competition behaviors. Glock and Kim (2015) studied a supplier’s integration strategy with multiple retailers' competition, in which the supplier can choose to cooperate with one retailer to operate an integrated channel. Zhang et al. (2020) discussed whether a green manufacturer opens a direct channel or not. These studies mainly focus on the structure of one manufacturer with multiple retailers or multiple manufacturers with one retailer. They have generated some important insights but do not focus on two entirely separate competing supply chains, in which each manufacturer has its exclusive retailer.

Competition between two separate supply chains was first studied by McGuire and Staelin (1983) in 1983. Anderson and Bao (2010) extended McGuire and Stalin’s model (1983) from two entirely separate chains to a more general context with arbitrary competing supply chains, and demonstrated that the underlying market shares play a very important role. Zhao and Shi (2011) studied two competing supply chains and focused on what supply chain structure and which contracting strategy a firm should choose. Chen et al. (2012) investigated pricing decisions in competing supply chains under different information structures. Yang et al. (2015) focused on channel strategy problem in supply chains with asymmetric competing products. Bian et al. (2016) analyzed a two-way information sharing problem in two competing supply chains under horizontal chain-to-chain competition. Li and Chen (2018) examined price and quality competition between two brands. Wu et al. (2018) investigated a horizontal Nash game and structure selection strategy in two competing dominant enterprises. They found that maintaining the same supply chain structure can generate dramatic spillover effect which benefits all the participants when they face horizontal competition. These studies mainly focus on channel structures with demand uncertainty or information sharing problems. There is also a stream considering retailers in two separate competing chains determine to cooperate. For instance, Wang and Liu (2019) focused on two shipping supply chains with the carrier of one chain contracting with not only the upstream port but also the carrier of the other chain. Wang et al. (2020) reviewed the literature on co-opetition relationships in transportation. However, these are different from the focus of our paper, which involves with green products and ordinary ones in two separate chains.

With respect to competition between GSC and traditional SC, Yalabik and Fairchild (2011) examined the effects of competitive pressure on firm investments in environmentally-friendly production. Liu et al. (2012) focused on the impact of consumers’ green awareness on supply chain players under competition. Their results show that an increase in consumer environmental awareness benefits retailers and manufacturers that have superior eco-friendly operations. Hafezalkotob (2017) developed competition and cooperation models for two GSCs under government financial intervention. Hafezalkotob (2018) examined competition of green supply chains and set up twelve models under government regulation policies. Zhang et al. (2014) considered the coordination strategy of GSC with hybrid production mode. Li and Li (2016) studied sustainable products under reverse chain-to-chain competition and derived equilibrium structures and found that vertical integration is Pareto optimal. Yang et al. (2017) focused on two separate supply chains and found that manufacturers’ horizontal cooperation will damage retailers’ profit and consumers’ welfare. Pu et al. (2018) constructed four scenarios, based on two separate competing supply chains, to investigate the effects of CGP on the market equilibrium of supply chains’ product selection strategy. Moradinasab et al. (2018) developed sustainable competitive petroleum supply chain model to minimize pollution while maximizing the profits and job creation. Jian et al. (2019) studied a multi-objective optimization model of green chain by considering environmental benefits. Nobari et al. (2019) introduced a multi-objective mathematical model to study the competition between two different supply chains. Yang et al. (2020) developed four game models for two competing supply chains, in which green marginal manufacturing cost, demand sensitivity of green level, and governmental interventions are considered. The above researches do not consider the truth that retailers with different types of products may cooperate to benefit themselves.

Most of the studies about cooperation and competition mainly focused on two chains of the same type with different brands or quality. They examined interactive effects of price and quality on the other chain’s demand and decisions, and illustrated whether decentralized or centralized structure is better for two chains, under different coordination contracts between upstream and downstream players. The study about cooperation and competition between GSC and ordinary SC is few in the literature. However, such mixed SCs do exist in practice. Yang et al. (2017) studied two separate competing chains between which two manufacturers may choose to cooperate. This paper will discuss the horizontal cooperation between retailers in two competing supply chains, and examine how CI and CGP impact decisions and profits. Moreover, the paper will try to generate Pareto optimal solution sets for retailers to cooperate and check whether manufacturers and whole supply chains can benefit from cooperation. That is, we will investigate whether it is good to implement GSC strategy if two retailers in different chains determine to cooperate.

## Problem descriptions and assumptions

### Problem descriptions

**Fig. 1.**
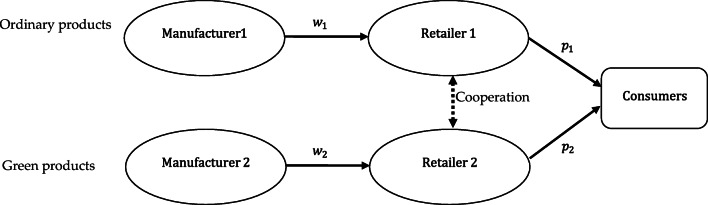
Competition between different supply chains

This paper considers two separate supply chains with competitive relationships, as shown in Fig. 1. The manufacturer in each chain is the leader and the retailer is the follower. However, the retailer is free to determine whether to cooperate with the other retailer, leading to different supply chain structures. Under non-cooperation, two retailers sell ordinary products and green products respectively; under cooperation, two retailers make centralized decisions and sell two types of products together. The dotted line in Fig. 1 indicates that there may be cooperation between retailers. Signs and notations involved in the paper are demonstrated in Table 1.

**Table 1:** Signs and notations

Signs	Notations
*a*	Market base
*μ*	Market share of ordinary products
*g*	Greenness of green products
*k*	*C*oefficient that represents for CGP
*r*	CI
*c*	R&D cost of green products
*D*_1_	Market demand for ordinary products
*D*_2_	Market demand for green products
${\pi}_{Mi}^{\ast \ast }$	Profit of manufacturer*i* (*i* = 1, 2)under non-cooperation
${\pi}_{Mi}^{\ast }$	Profit of manufacturer*i* (*i* = 1, 2)under cooperation
${\pi}_{Ri}^{\ast \ast }$	Profit of retailer*i* (*i* = 1, 2)under non-cooperation
${\pi}_{Ri}^{\ast }$	Profit of retailer*i* (*i* = 1, 2)under cooperation
${\pi}_R^{\ast }$	Total profit of two retailers under cooperation
Δ*π*_1_	Profit increment of the entire ordinary product supply chain
Δ*π*_2_	Profit increment of the entire green product supply chain

### Assumptions

To clarify the models, solutions, and numerical analysis, we give assumptions as below.

#### Assumption 1.

#### Symmetrical information and leader-follower relationship

Market information is completely symmetrical, and manufacturers and retailers are rational to gain much more profit. This paper considers competition and substitution between ordinary and green products. In order to survive, it is an inevitable choice for participants to understand their competitors and products well. Moreover, advanced payment, data technology, green promotion, and government policies lead to symmetrical information.

Competition between ordinary and green products is also the competition between two types of manufactures, which dominate its own chain. The decision process in each chain is essentially a Stackelberg game. The manufacturer as the leader first determines the wholesale price and the greenness, and the retailer then maximizes its own profits as a follower (Jamali and Rasti-Barzoki 2018; Yang et al. 2020; Meng et al. 2021).

#### Assumption 2.

#### Cost function of green products

Cost function of green products has a few forms, such as *c* = *C* − *γd*, where *C* is initial unit cost, *γ* is the learning coefficient and *d* is the accumulated sales volume of green products (Yenipazarli and Vakharia 2015). Zhu and He (2017) marked the cost as *fg*^2^. Hong and Guo (2019) used *αg*^2^ to describe it, which is the same as Zhu and He (2017). Jamali and Rasti-Barzoki (2018) used *μg*^2^/2 to sign it. Murali et al. (2019) denoted it as *g*^2^. Yang et al. (2020) considered both fixed cost *μg*^2^/2 and variable cost *cg*, and the latter part indicates a marginal cost *c* when the greenness is improved by one unit. Meng et al. (2021) signed the green cost with a constant parameter. Based on these researches, we refer to Murali et al. (2019) and use a simple green cost function of *g*^2^ because our focus is how CI and CGP impact optimal decisions, and green cost function’s form is not the point.

#### Assumption 3.

#### Demand functions

This article examines green and non-green products, so that the demand function should be discussed with two parts. There are also many related studies. Meng et al. (2021) described the demand for green and non-green products as *q*_*g*_ = (1 − *ρ*)*α* − *βp*_*g*_ + *γp*_*n*_ + *ηg* and *q*_*n*_ = *ρα* − *βp*_*n*_ + *γp*_*g*_ + (1 − *η*)*g*, respectively. Yang et al. (2020) used *q*_*n*_ = *ρa* − *p*_*n*_ + *bp*_*g*_ − *λ*_1_*g* and *q*_*g*_ = (1 − *ρ*)*a* − *p*_*g*_ + *bp*_*n*_ + *λ*_2_*g* to denote the demand for two types of products, respectively. Zhu and He (2017) introduced demand functions as *q*_1_ = *a* − *p*_1_ + *s*_*p*1_*p*_2_ + *b*(*g*_1_ − *s*_*g*1_*g*_2_) and *q*_2_ = *a* − *p*_2_ + *s*_*p*2_*p*_1_ + *b*(*g*_2_ − *s*_*g*2_*g*_1_); if we let *g*_1_ = 0 and denote *g*_2_ as *g*, the functions can be rewritten as *q*_*n*_ = *a* − *p*_1_ + *s*_*p*1_*p*_2_ − *bs*_*n*_*g* and *q*_*g*_ = *a* − *p*_2_ + *s*_*p*2_*p*_1_ + *bs*_*g*_*g*. Jamali and Rasti-Barzoki (2018) considered them as *q*_*n*_ = *ρα* − *βp*_*n*_ + *γp*_*g*_ − *λ*_1_*g* and *q*_*g*_ = (1 − *ρ*)*α* − *βp*_*g*_ + *γp*_*n*_ + *λ*_2_*g*. These functions give us meaningful insights and can help us to propose our own green and non-green demand functions. We also reviewed those in McGuire and Staelin (1983), which are as follows

$$ {q}_1=\mu S\left[1-\frac{\beta }{1-\theta }{p}_1+\frac{\beta \theta}{1-\theta }{p}_2\right], $$

$$ {q}_2=\left(1-\mu \right)S\left[1-\frac{\beta }{1-\theta }{p}_2+\frac{\beta \theta}{1-\theta }{p}_1\right]. $$

However, the products discussed in McGuire and Staelin (1983) are not green and non-green ones. Based on these demand functions and the practical statement in Introduction, we have the demand like below

1 $$ {D}_1= a\mu -\frac{p_1}{1-r}+\frac{rp_2}{1-r}- kg $$

2 $$ {D}_2=a\left(1-\mu \right)-\frac{p_2}{1-r}+\frac{rp_1}{1-r}+ kg $$

As shown above, market demand depends on its own product price, competitive product price, CI, CGP, and greenness. Different from Zhu and He (2017), Jamali and Rasti-Barzoki (2018), Yang et al. (2020), and Meng et al. (2021), we assume the impact of greenness on green products’ demand is the same as that on ordinary products’ demand. Most of the existing researches believed that the impact of greenness on green demand is always greater than that on ordinary ones. That is, for every additional unit of greenness, the increase in demand for green products is greater than the decline in ordinary products. However, Ginsberg and Bloom (2004) pointed out that in dealing with green market-related issues, researchers and managers cannot ignore that consumers do not want to obtain green benefits at the expense of the original convenience, quality, and other commodity attributes. As stated in Damigos et al. (2020), green refrigerators are not so easy even hard to sell and there are some other factors influencing demand apart from CGP, such as currency tag and heterogeneity of consumer behavior. All of these tell us that the impact on green products may not be greater than that on ordinary ones. Refer to Murali et al. (2019), *q*_*H*_ = 1/2 − *p*_*H*_ + (*p*_*L*_ − *p*_*H*_) + *β*(*μ*_*H*_*g*_*H*_ − *μ*_*L*_*g*_*L*_) and *q*_*L*_ = 1/2 − *p*_*L*_ + (*p*_*H*_ − *p*_*L*_) + *β*(*μ*_*L*_*g*_*L*_ − *μ*_*H*_*g*_*H*_) are the demand functions. If we let *g*_*L*_ = 0 and denote *g*_*H*_ as*g*, there will be *q*_*g*_ = 1/2 − *p*_*g*_ + (*p*_*n*_ − *p*_*g*_) + *βμ*_*g*_*g* and*q*_*n*_ = 1/2 − *p*_*n*_ + (*p*_*g*_ − *p*_*n*_) − *βμ*_*g*_*g*, which means the impacts are the same. This is consistent with our demand functions, but we assume the market base of two types of products is different and the price substitutability *r* is not fixed but varying.

#### Assumption 4.

#### CI and product substitutability

As stated in Eqs. (1) and (2), the ratio of 1/(1 − *r*) to *r*/(1 − *r*) is *r*, which refers to the ratio of market demand with respect to the competitor’s price to change of market demand with respect to own price. Thus, we denote *r *as CI between two chains. When *r* = 0, the demands are independent, and each chain is a monopolist. Product substitutability increases with *r* until as *r* approaches 1, in which situation the products are maximally substitutable. This prediction is the same as in McGuire and Staelin (1983). However, our paper shows that optimal results and equilibria depend not only on product difference but also on CGP.

#### Assumption 5.

#### Market share and fixed production cost

*μ* is the market share of ordinary products and reflects consumer loyalty to ordinary products (Jamali and Rasti-Barzoki 2018; Yang et al. 2020; Meng et al. 2021). Thus, 1 − *μ* is that of green products and reflects consumer loyalty to green ones.

The fixed production cost that not related with greenness is assumed to be zero (Zhu and He 2017) and this does not change the qualitative findings in our paper. This helps the optimal decision results to be presented much more clearly.

## Models and solutions

According to the assumptions discussed in the Section “Assumptions,” we can obtain two manufacturers’ profit functions as below.

3 $$ {\pi}_{M1}={w}_1\left( a\mu -\frac{p_1}{1-r}+r\frac{p_2}{1-r}- kg\right) $$

4 $$ {\pi}_{M2}={w}_2\left[a\left(1-\mu \right)-\frac{p_2}{1-r}+r\frac{p_1}{1-r}+ kg\right]-{g}^2 $$

The profits functions of ordinary product retailer and green product retailer are given by:

5 $$ {\pi}_{R1}=\left({p}_1-{w}_1\right)\left( a\mu -\frac{p_1}{1-r}+r\frac{p_2}{1-r}- kg\right) $$

6 $$ {\pi}_{R2}=\left({p}_2-{w}_2\right)\left[a\left(1-\mu \right)-\frac{p_2}{1-r}+r\frac{p_1}{1-r}+ kg\right] $$

### Supply chain game models without cooperation

If there is no cooperation between two chains, under Stackelberg game, the two retailers will maximize their own profits, respectively. It is easy to find that both their second-order derivatives are less than zero and both profit functions can be optimized. When we make the first-order derivatives equal to zero, the optimal prices are as follows:

7 $$ {p}_1=\frac{2{w}_1+{rw}_2+2 a\mu -2 kg+ a r-{ar}^2-{kgr}^2-3 a\mu r+3 gkr+ a\mu {r}^2}{4-{r}^2} $$

8 $$ {p}_2=\frac{rw_1+2{w}_2-2 a\mu +2 kg+2a-2 a r+{kgr}^2+3 a\mu r-3 kgr- a\mu {r}^2}{4-{r}^2} $$

Then we substitute the price solutions above into profit functions of manufacturers, and then maximize them to get optimal solutions, as described in Theorem 1.

Theorem 1.

Ordinary manufacturer’s profit changes concavely under non-cooperation. Green manufacturer’s profit can be maximized if $0\le k<2\sqrt{\left(2+r\right)\left(2-{r}^2\right)/\left(2-r\right)\left(1-r\right)}$, and other related solutions are described in Table 2.

**Table 2:** Optimal solutions and profits of two chains under non-cooperation between retailers

Decision variables	Supply chain 1 (ordinary products)	Supply chain 2 (green products)
*w*^∗∗^	$\frac{a\left(r-1\right)\left(r+2\right)C}{\left(2{r}^2+r-4\right)E}$	$\frac{2a\left(r-1\right)\left(r+2\right)G}{\left(2{r}^2+r+4\right)E}$
*g*^∗∗^	---	$\frac{ak\left(r-1\right)G}{\left(2{r}^2+r-4\right)E}$
*p*^∗∗^	$\frac{2 aJC}{DE}$	$\frac{4 aJG}{DE}$
*D*^∗∗^	$\frac{a\left({r}^2-2\right)C}{DE}$	$\frac{2a\left({r}^2-2\right)G}{DE}$
${\pi}_M^{\ast \ast }$	$\frac{a^2\left({r}^2-2\right)\left(r-1\right)\left(r+2\right){C}^2}{\left(2{r}^2+r-4\right){DE}^2}$	$-\frac{a^2\left(r-1\right){FG}^2}{\left(r-2\right){\left(2{r}^2+r-4\right)}^2{E}^2}$
${\pi}_R^{\ast \ast }$	$\frac{4{a}^2{\left({r}^2-2\right)}^2\left(1-r\right){G}^2}{D^2{E}^2}$	$\frac{a^2{\left({r}^2-2\right)}^2\left(1-r\right){C}^2}{D^2{E}^2}$

### Supply chain game models under cooperation

When two retailers determine to cooperate, we will consider them as a whole system and maximize the total profit as below.

9 $$ \mathit{\operatorname{Max}}\underset{p_1,{p}_2}{\pi_R}=\left({p}_1-{w}_1\right)\left( a\mu -\frac{p_1}{1-r}+r\frac{p_2}{1-r}- kg\right)+\left({p}_2-{w}_2\right)\left[a\left(1-\mu \right)-\frac{p_2}{1-r}+r\frac{p_1}{1-r}+ kg\right] $$

Optimal solutions can be found in Theorem 2.

#### Theorem 2.

When two retailers cooperate horizontally, total retail profit function changes concavely with*p*_1_and*p*_2_, and has extreme values.

The optimal prices can be obtained by making the first derivatives be zero, and the solutions are as follows:

10 $$ {p}_1=\frac{w_1+ a\mu - kg+ a r+{rw}_1- a\mu r+ kgr}{2\left(r+1\right)} $$

11 $$ {p}_2=\frac{w_2- a\mu + kg- a r+{rw}_1+ a\mu r- kgr}{2\left(r+1\right)} $$

Substituting the above results into the manufacturers’ profit functions, we can obtain the solutions in Theorem 3 and Table 3.

**Table 3:** Optimal solutions and profits of supply chains under cooperation between retailers

Decision variables	Supply chain 1 (ordinary products)	Supply chain 2 (green products)
*w*^∗^	$\frac{a\left(r-1\right)A}{\left(r-2\right)B}$	$\frac{4a\left(r-1\right)\left(\mu r-2\mu +2\right)}{\left(r-2\right)B}$
*g*^∗^	—	$\frac{ak\left(r-1\right)\left(\mu r-2\mu +2\right)}{\left(r-2\right)B}$
*p*^∗^	$\frac{aH}{2\left(-{r}^2+r+2\right)B}$	$\frac{- aI}{2\left(-{r}^2+r+2\right)B}$
*D*^∗^	$-\frac{aA}{2\left(r-2\right)B}$	$-\frac{2a\left(\mu r-2\mu +2\right)}{\left(r-2\right)B}$
${\pi}_M^{\ast }$	$-\frac{a^2\left(r-1\right){A}^2}{2{\left(r-2\right)}^2{B}^2}$	$-\frac{a^2\left(r-1\right)\left({k}^2r-{k}^2+8\right){\left(\mu r-2\mu +2\right)}^2}{{\left(r-2\right)}^2{B}^2}$
${\pi}_R^{\ast }$	$\frac{a^2K}{4\left(r+1\right){\left(r-2\right)}^2{B}^2}$	

#### Theorem 3.

When two retailers cooperate, ordinary manufacturer’s profit is still a concave function of wholesale price. However, green manufacturer’s profit can be maximized only if $0\le k\le 2\sqrt{2/\left(1-r\right)}$.

Compared with the solutions under non-cooperation between retailers, the optimal results are different and can be found in Proposition 1.

#### Proposition 1.

When retailers cooperate horizontally, the greenness, retail price, wholesale price, and market demand of green products all change positively with CGP, but those of ordinary ones all change reversely with CGP.

Proposition 1 shows that the more sensitive consumers are to greenness, the easier it is for market demand to increase as a result, and the easier it is for manufacturers to increase the greenness of their products. The rising tide will lead to higher retail and wholesale prices. On the contrary, demand for ordinary products has shrunk due to CGP, and wholesale and retail prices have to be lowered accordingly. Nevertheless, if there is no cooperation between retailers, the relevant results need to be further analyzed through numerical experiments.

#### Proposition 2.

When retailers cooperate, if $0\le k<2\sqrt{\left(2-r\right)/\left(1-r\right)}$, green manufacturer’s profit increases with CGP; if $2\sqrt{\left(2-r\right)/\left(1-r\right)}\le k\le 2\sqrt{2/\left(1-r\right)}$, it decreases with CGP.

Proposition 2 shows that there is a positive correlation between the profit changes of green manufacturer and CGP if CGP is less than a critical value. This is also the driving force for manufacturers to become green. However, if consumers are too sensitive to greenness so that CGP is bigger than the critical value, green manufacturer’s profit will decrease instead. When retailers do not conduct horizontal cooperation, whether there is such a correlation, it needs to be further discussed through numerical experiments.

### Decision comparisons between cooperation and non-cooperation

It is clear from the above analysis that there will be differences between cooperation and non-cooperation; in particular, wholesale prices, retail prices, greenness of green products, demands, and manufacturers’ profits are shown Table 4. Whether the differences are positive or not depends on some coefficients, which can be found in Table 4.

**Table 4:** Differences of optimal solutions between cooperation and non-cooperation

Differences	Supply chain 1 (ordinary products)	Supply chain 2 (green products)
Δ*g* = *g*^∗^ − *g*^∗∗^	**-**	$\frac{ak\left(1-r\right)\left[ LME+\left(2-r\right) BG\right]}{MBE\left(2-r\right)}$
Δ*w* = *w*^∗^ − *w*^∗∗^	$\frac{ak\left(1-r\right)\left[ LME+\left(4-{r}^2\right) BG\right]}{MBE\left(2-r\right)}$	$\frac{2a\left(1-r\right)\left[2 LME+\left(4-{r}^2\right) BG\right]}{\left(2-r\right) MBE}$
Δ*p* = *p*^∗^ − *p*^∗∗^	$\frac{a\left[ HDE-4 NBCJ\right]}{2 NBDE}$	$\frac{-a\left[ DEI+8 NBGJ\right]}{2 NBDE}$
Δ*D* = *D*^∗^ − *D*^∗∗^	$\frac{a\left[ ADE+2\left({r}^2-2\right)\left(r-2\right) BC\right]}{2\left(2-r\right) BDE}$	$\frac{2a\left[ LDE+\left(2-{r}^2\right)\left(2-r\right) BG\right]}{\left(2-r\right) BDE}$
Δ*π*_*M*_ = *π*_*M*_^∗^ − *π*_*M*_^∗∗^	$\frac{a^2\left(1-r\right)\left[{MA}^2{DE}^2+2\left({r}^2-4\right)\left({r}^2-2\right)\left(r-2\right){B}^2{C}^2\right]}{2{\left(r-2\right)}^2{MB}^2{DE}^2}$	$\frac{a^2\left(1-r\right)\left[\left({k}^2r-{k}^2+8\right){(LME)}^2+\left(2-r\right){B}^2{FG}^2\right]}{{\left[\left(r-2\right) MBE\right]}^2}$

#### Proposition 3.

If *μ* ≤ (8 − 3*r*^2^)/(4 − *r* − 2*r*^2^) and $k\ge 2\sqrt{\left(2+r\right)/\left(1-r\right)}$, there will be Δ*g* < 0, Δ*w*_1_ < 0,, Δ*w*_2_ < 0,, and Δ*D*_2_ < 0.

Proposition 3 tells us that when market share of ordinary products is smaller than a certain value and CGP exceeds a certain threshold, greenness of green products, wholesale price of both ordinary and green products, and demand for green products will shrink due to cooperation. This means that when market share of ordinary products is a little small and consumers are quite sensitive to green products, cooperation will dilute the value of green products and bring falling prices and shrinking demand. Under this scenario, independent operation may be the better choice.

Nevertheless, we need to confirm whether both retailers are willing to cooperate. The premise of the cooperation is that the profits of both retailers can be improved and they can achieve a win-win situation. We suppose the allocation of total retail profit is given as follows:

12 $$ {\prod}_{R1}^{\ast }=\lambda {\prod}_R^{\ast } $$

13 $$ {\prod}_{R2}^{\ast }=\left(1-\lambda \right){\prod}_R^{\ast } $$

Coefficient *λ* in above expressions changes in the interval [0, 1]. Only when the profits of both retailers are improved due to cooperation, they are willing to cooperate. Thus, there should be the following expressions:

14 $$ \lambda {\prod}_R^{\ast}\ge {\prod}_{R1}^{\ast \ast } $$

15 $$ \left(1-\lambda \right){\prod}_R^{\ast}\ge {\prod}_{R2}^{\ast } $$

Then, we can get an exact range as *λ*_min_ ≤ *λ* ≤ *λ*_max_, *λ*_min_ and *λ*_max_ can be obtained as follows:

16 $$ {\lambda}_{\mathrm{min}}=\frac{16{\left({r}^2-2\right)}^2\left(1-{r}^2\right){\left(r-2\right)}^2{B}^2{G}^2}{D^2{E}^2K} $$

17 $$ {\lambda}_{\mathrm{max}}=1-\frac{4{\left({r}^2-2\right)}^2\left(1-{r}^2\right){\left(r-2\right)}^2{B}^2{C}^2}{D^2{E}^2K} $$

## Numerical analysis

To analyze the impacts of CGP and CI on optimal price decisions, greenness, demands, and profits, we change coefficients *a*, *μ*, *k*, and *r* systematically, and find that the general trends of prices with CI or CGP are basically the same, so do greenness, demands, and profits. Thus, we just choose some typical examples to discuss. For instance, we assign *a* = 10 and *μ* = 0.4 to see how prices and other results change with CGP and CI. If CGP needs to be static, we will assign *k* = 2. If CI needs to be static, we will assign *r* = 0.5. Regarding the market share, the existing researches often set it around 0.4 in numerical experiments, such as Hafezalkotob (2015), Hafezalkotob (2017), Yang et al. (2020), and Meng et al. (2021). More detailed analyses are given as follows.

### Influences on optimal decisions and results of CI and CGP

Three theorems and three propositions all tell us that CI and CGP play very important roles in obtaining optimal decisions and profits. In this section, we will illustrate how price strategy, greenness, demands, and profits change with CI and CGP. Those under both cooperation and non-cooperation are listed with figures and analysis.

#### Trends of retail prices

**Fig.2:**
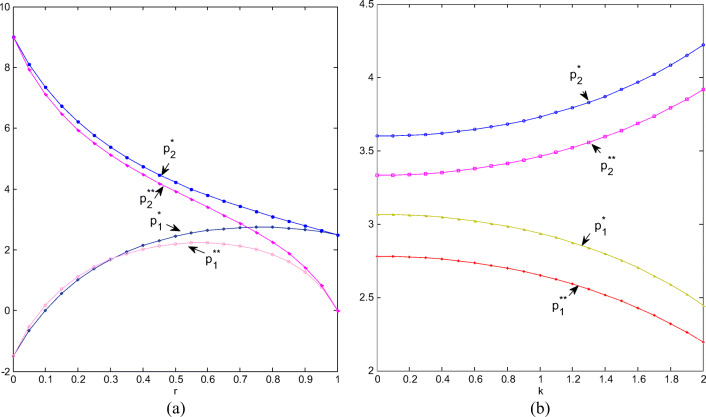
(**a**) Retail prices change with r; (**b**) Retail prices change with k.

Firstly, we assign *k* = 2 to illustrate how retail prices change with *r*, as shown in Fig. 2a. Regardless of cooperation or non-cooperation, the retail price of green products is higher than that of ordinary products. Green products’ retail price under cooperation is higher than that under non-cooperation; but for ordinary ones, when CI is weak, the price is higher under non-cooperation; when CI is stronger than 0.3, the price under cooperation is higher. When CI approaches 1, the corresponding prices of green and ordinary products will approach the same, but the price under cooperation will be higher than that under non-cooperation. The fiercer the competition between two chains means the smaller the difference between green and ordinary products and the stronger the substitution. Therefore, the retail prices of two types of products tend to be equal when CI approaches 1. The cooperative price is generally higher than the non-cooperative one because retail integration probably reduces marketing, channel operation, and advertising costs. Nevertheless, when CI equals zero, two types of products cannot replace each other at all. The retail price of green products is much higher than that of ordinary products. The latter will be unsalable, and cooperation or non-cooperation will have no effect on retail prices.

Secondly, we assign *r* = 0.5 to illustrate how retail prices change with *k*, as shown in Fig. 2b. The retail prices of green and ordinary products have the opposite trend with *k*. The more sensitive the market is to product greenness, the higher the retail price of green products, and the lower the retail price of ordinary products. Regardless of whether two retailers cooperate or not, the retail price of green products is always higher than that of ordinary products. Regardless of whether it is a green product or an ordinary one, the retail price under cooperation is always higher than the corresponding price under non-cooperation. The sensitivity of the market to green products has caused competition between green and ordinary products, which will inevitably lead to an increase in the price of the former and a decrease in the latter. Whether it is for green or ordinary products, cooperation will help increase retail prices.

#### Trends of wholesale prices

**Fig.3:**
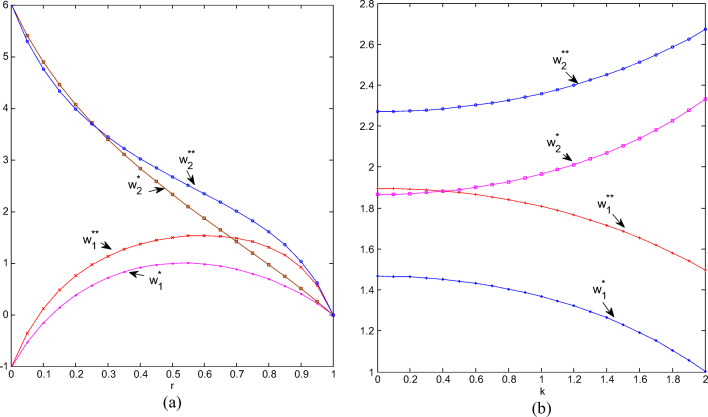
(**a**) Wholesale prices change with r; (**b**) Wholesale prices change with k.

Firstly, we assign *k* = 2 to illustrate how wholesale prices change with *r*, as shown in Fig. 3a. For ordinary products, the wholesale price under non-cooperation is higher than that under cooperation. For green products, when CI is weak, the wholesale price of cooperation is higher, but when CI exceeds 0.24, the wholesale price of non-cooperation is higher. Regardless of cooperation or not, the wholesale price of green products is always higher than that of ordinary products and this is closely related to the additional production costs of green products. When CI tends to 1, there exists an absolute substitute between green and ordinary products, and all wholesale prices tend to be equal, regardless of cooperation or non-cooperation. This is quite different from the situation when CI is zero. In this scenario, green and ordinary products are completely irreplaceable, the wholesale price of the former is much higher than the latter, and ordinary products’ wholesale price is even negative. At this time, whether there is cooperation between retailers has no effect on the wholesale price. This means that when there is an absolute difference between the two types of products, the manufacturer only needs to pay attention to the greenness of the product when setting the wholesale price, and does not need to pay attention to retailers’ cooperation behavior.

Secondly, we assign *r* = 0.5 to illustrate how retail prices change with *k*, as shown in Fig. 3b. The more sensitive the market is to greenness, the higher the wholesale price of green products and the lower the wholesale price of ordinary products. The wholesale price of green products is always higher than that of ordinary products, which has nothing to do with whether the retailer cooperates. Regardless of whether it is for green or ordinary products, the wholesale price of non-cooperation is higher than that of cooperation.

#### Trends of greenness of green products

**Fig.4:**
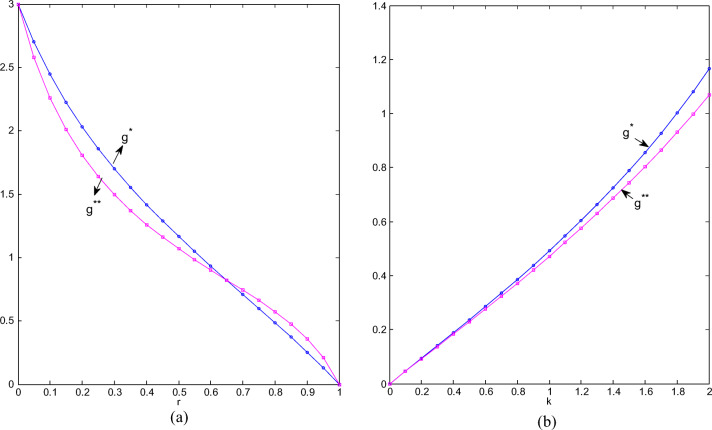
(**a**) Greenness changes with r; (**b**) Greenness changes with k.

As shown in Fig. 4a, whether two chains cooperate or not, the stronger the competition, the lower the greenness. When the competition intensity is at the extreme of 0 or 1, cooperation or not has no effect on the greenness level, but when CI is 0, the greenness of green products is the highest. When it is 1, the greenness level is the lowest, and green products are reduced to normal ones. When CI is lower than 0.65, the greenness level of cooperation is higher than that of non-cooperation; otherwise, the greenness level of non-cooperation will be higher. This means that when CI is relatively strong and the two types of products are highly substitutable, non-cooperation is conducive to improving the greenness of green products. Conclusively, when the competition between two chains gradually intensifies, if the retailers do not cooperate, the green manufacturer will reduce the greenness level relatively quickly at first, and then hesitate so that the speed will slow down; if two retailers cooperate, the green manufacturer will reduce the greenness level with a relatively high speed, and it seems that the cooperation has exacerbated the confusion between green products and ordinary products.

As shown in Fig. 4b, the more sensitive the market is to greenness, the higher the greenness; the greenness under cooperation is higher than that under non-cooperation. When the market is not sensitive enough to greenness, the greenness of cooperation and non-cooperation tends to be the same; when the market is more sensitive to it, cooperation between retailers will bring about a greater increase in the greenness of green products.

#### Trends of market demands

**Fig.5:**
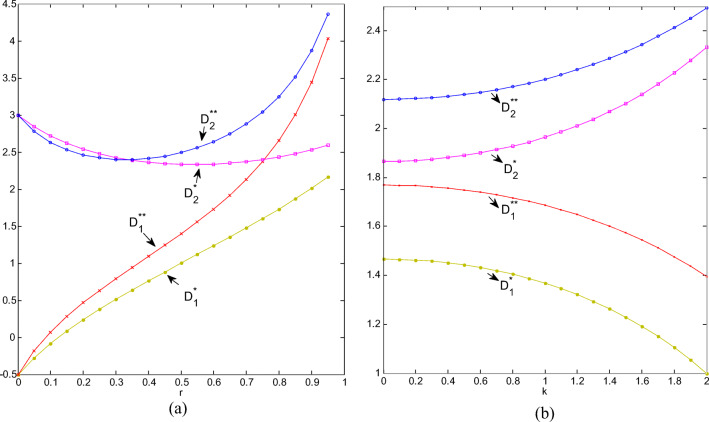
(**a**) Demands change with r; (**b**) Demands change with k.

When the competition intensity is 0, market demands separately corresponding to cooperation and non-cooperation are the same; when the competition intensity is 1, they are the same as well. For ordinary products, the market demand under non-cooperation is higher than that under cooperation, and as CI becomes stronger, the difference in demand between non-cooperation and cooperation increases. For green products, when CI is less than 0.34, the market demand under cooperation is greater, and when CI is more than 0.34, the corresponding market demand under non-cooperation is greater. In general, when the substitution between green and ordinary products gradually increases, cooperation between retailers will inhibit market demand. However, regardless of cooperation or not, the market demand for green products is greater than that for ordinary products, especially when two types of products cannot substitute for each other at all; when the substitution becomes stronger, the market demand for green products and ordinary products gradually become consistent, as shown in Fig. 5a.

As shown in Fig. 5b, when consumers become more and more sensitive to greenness, the market demand for green products gradually increases, and the market demand for ordinary ones gradually declines. Regardless of whether there is cooperation between retailers, the market demand for green products is always greater than that for ordinary products, and as the sensitivity increases, the market demand for two types of products is increasingly different. Regardless of green or ordinary products, the demand in the non-cooperative market is greater than that during cooperation, which further shows that cooperation is not conducive to the expansion of demand.

#### Trends of profits

**Fig.6:**
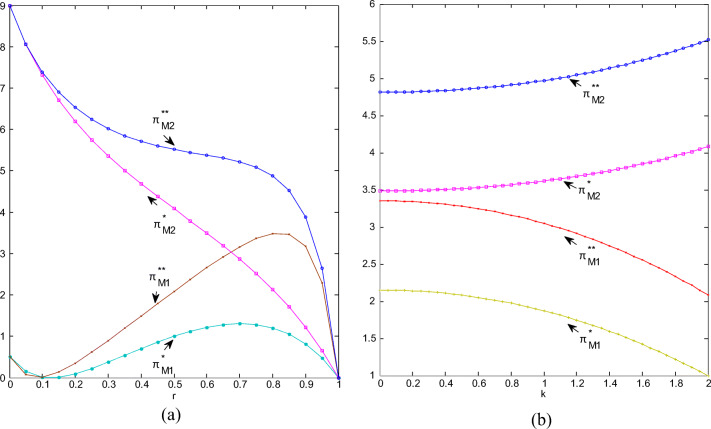
(**a**) Manufacturers’ profits change with r; (**b**) Manufacturers’ profits change with k.

As competition intensifies and the substitution of green and ordinary products increases, the profits of green product manufacturers generally show a downward trend; when there is no cooperation, because manufacturers hesitate to reduce the degree of greenness, the decline in their profits is also relatively flat as CI is neither too weak nor too strong. When competition gradually tends to 1, the profit decline is almost cliff-like and quickly drops to zero; see Fig. 6a. When working together, the process of green manufacturers’ reduction of greenness is almost linear, and their profit decline also shows a similar trend. For ordinary products, regardless of cooperation or not, in the process of increasing competition, the manufacturer’s profits show multiple inflection points; when there is no cooperation, the profit reaches a maximum value when CI is 0.82, and the maximum value during cooperation is reached when CI is equal to 0.72. Generally, for both types of products, the manufacturer’s profit under non-cooperation is greater than the corresponding profit under cooperation. In both cases, as CI increases, the profit margin between non-cooperation and cooperation increases first and then decreases rapidly. Regardless of cooperation or not, green product manufacturers’ profit is greater than that of ordinary product manufacturer, especially when the substitution is weak, the former is much greater than the latter. In other words, when green products have an absolute competitive advantage, the profit disparity among manufacturers is considerably large.

Regardless of cooperation or not, the profits of green manufacturers continue to increase convexly as the market becomes more and more sensitive to the degree of greenness, while the profits of ordinary manufacturers keep decreasing concavely. Regardless of the type of products, the profit of the manufacturer during non-cooperation is obviously greater than the profit during cooperation, which shows that the cooperation between retailers has caused manufacturers’ profit to decline, and the manufacturer would be disgusted with the cooperation between retailers. The profit of green manufacturer is always greater than that of ordinary manufacturer, and as consumers become more sensitive to greenness, the profit difference between two types of manufacturers is getting larger and larger; see Fig. 6b. We can conclude that consumers’ preference for green products puts ordinary product manufacturers facing greater pressure to survive, and they will try to improve their product competitiveness for further development, such as introducing green materials and processes to promote the green development of whole industry. Therefore, strengthening consumers’ awareness of environmental protection and enhancing their sensitivity to green products are conducive to promoting the development of green supply chains.

**Fig.7:**
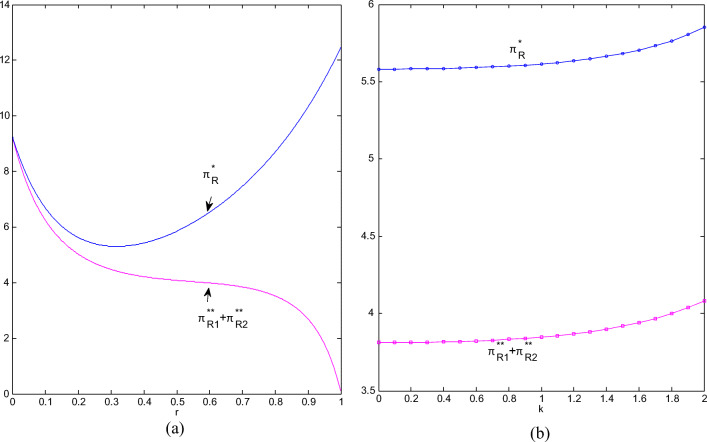
(**a**) Total profits of retailers change with r; (**b**) Manufacturers’ profits change with k.

It can be seen from Fig. 7a and Fig. 7b that the total retail profit has increased after the cooperation. The profit shows a convex trend as the competition intensifies. It drops to a minimum when CI is about 0.3, and then continues to rise until it reaches the maximum when the competition is sufficient. The total profit under non-cooperation is first with a rapid decline and then with an ease; when CI is greater than 0.8, it declines quickly. The more intense the competition, the greater the room for profit improvement after cooperation. The more sensitive the market is to greenness, the higher the total retail profit will be; the total profit after cooperation is much greater than that before cooperation, but the profit improvement brought about by cooperation is relatively stable with the sensitiveness to greenness.

### Pareto zones for retailers to cooperate

**Fig.8:**
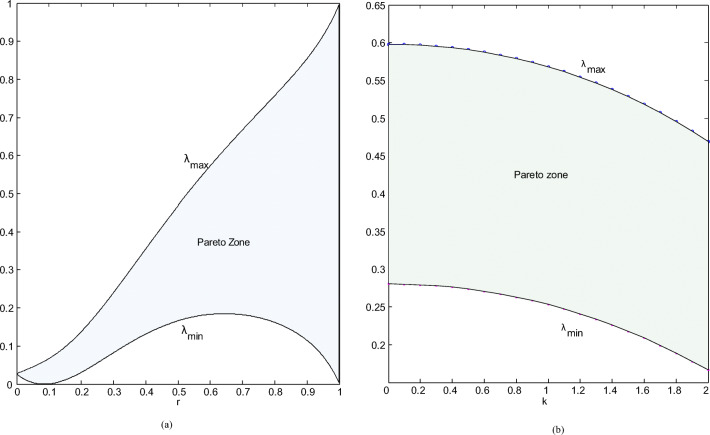
(**a**) λ changes with r; (**b**) λ changes with k.

The premise of cooperation between retailers is that the profits of both parties can be improved. The distribution coefficient *λ* usually has a minimum and a maximum, as shown in “Decision comparisons between cooperation and non-cooperation.” Figure 8 a shows that as the competition between the two chains gradually increases, the maximum value increases almost linearly, while the minimum value has multiple inflection points. The maximum value is obtained at 0.65, and the minimum value is obtained at 0.1 and 1. If (*λ*_max_ − *λ*_min_) × 100% is used to represent the probability of two retailers’ profit to achieve Pareto improvement, it is not difficult to find that the probability continues to increase as competition intensifies, and the distribution coefficient *λ* can take almost all values on the interval [0.1]. This means that when CI is fierce, the possibility that retailers can achieve profit improvement through cooperation is close to 100%, and the cooperation between the two is bound to bring a win-win situation.

As the market’s sensitivity to greenness has gradually increased, the probability of retailers’ willingness to cooperate has not changed significantly, but the maximum and minimum values ​​are both concave. For example, if consumers are completely insensitive to greenness, the distribution coefficient’s minimum and maximum values ​​are 0.28 and 0.58, respectively; when the sensitivity continues to increase to 2, the minimum and maximum values ​​of *λ* are 0.15 and 0.48, respectively, and the interval length of *λ* has been stable at about 0.3; that is, the probability of achieving a Pareto-optimal is approximately 30%. We can conclude that consumers’ sensitivity to greenness does not necessarily prompt retailers to participate under cooperation.

A more important question is how to get the exact value of coefficient *λ* under cooperation. It is probably relevant with the two retailers’ bargaining powers and risk preferences. That how to get the exact value is omitted here for the details are similar to that discussed by Shang and Yang (2015).

### Profit increment comparison between two supply chains

**Fig.9:**
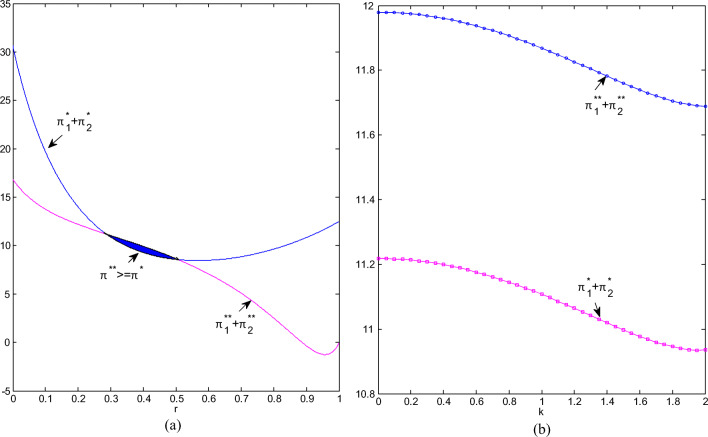
(**a**) Total profits of two chains change with r; (**b**) Total profits of two chains change with k.

If total profit of two supply chains is taken as the overall effect index produced by the cooperation between retailers, the entire supply chain system will benefit when CI is weak or strong. For example, it can be seen from Fig. 9a that when CI is lower than 0.29 or higher than 0.51, the total profit under cooperation is significantly greater than that under non-cooperation. Nevertheless, cooperation is not more beneficial in all situations. For example, when CI is greater than 0.29 and less than 0.51, non-cooperation is more conducive to the development of entire supply chain system. This tells us that when the substitution between green and ordinary products is not obvious or quite obvious, cooperation between retailers is superior to non-cooperation; when the substitution between the two gradually just tends to be obvious, independent operations between retailers are more effective and conducive to the development of the system.

If the state of competition is stable and consumers’ sensitivity to greenness gradually becomes stronger, regardless of cooperation or not, the profit of entire supply chain competition system will be in a slow downward trend, but the profit value corresponding to non-cooperation is always significantly greater than that of cooperation (see Fig. 9b). Consumers’ sensitivity to green products tells that supply chain system needs to increase investment in R&D and production, resulting in a decline in total profits. This is also the right reason why the brown chain still exists. The competition between manufacturing chains has not completely transformed into that between pure green chains.

**Fig. 10:**
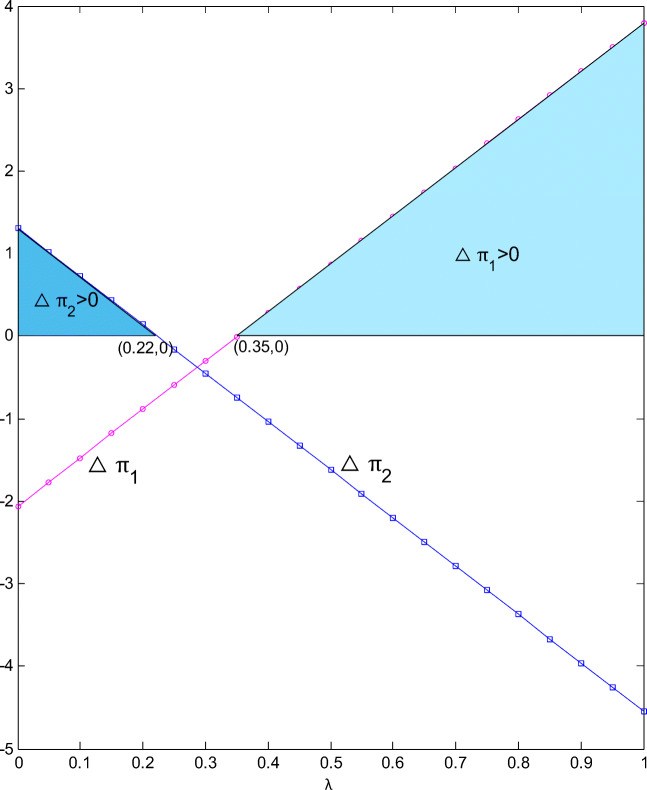
Profit increment changes with λ

The profit change of entire supply chain system is not affected by the profit distribution coefficient *λ*, but the change of each chain’s own profit is closely related to how total retail profit under cooperation is distributed. Figure 10 shows that it is feasible for two retailers to achieve a win-win situation when *r* = 0.5 and *k* = 2, but there seems to be no possibility for both chains to achieve a win-win situation based on the cooperation between retailers; when *λ* is less than 0.22, the overall profit of green supply chain is improved, but the ordinary product chain’s profit is severely damaged; when *λ* is greater than 0.35, the overall profit of ordinary chain is improved, but the green chain is seriously damaged; when *λ* changes on the interval [0.22, 0.35], the profits of both chains are damaged due to the cooperation between retailers. But this also tells us that both chains can achieve their own improvement by attracting the other chain’s retailer to participate in cross-chain cooperation. Cooperation itself will bring down the manufacturer’s profit, but if the profit growth of a chain is positive, it means that its retailer’s profit improvement is far greater than the manufacturer’s profit loss. After the loss is balanced, there is still a surplus (see Table 5 and Table 6). The retailer may compensate the manufacturer so that it can also benefit from cooperation, and then the manufacturer will no longer mind the retailer’s cross-chain cooperation. Based on this logic, referring to Figure 10, it seems that ordinary product chain has more opportunities. If its negotiating ability can make *λ* greater than 0.35 or even close to 1, the profit improvement of the chain is relatively considerable, which will realize a win-win membership easily. Especially for brand manufacturers, they impress consumers much, and encouraging retailers to cooperate across chains and formulating relevant profit transfer clauses can also be a way of survival, while facing the competition of green products.

**Table 5:** Profit increments of whole supply chains (*k* = 2)

*r*	Profit increments	*λ*										
		0	0.1	0.2	0.3	0.4	0.5	0.6	0.7	0.8	0.9	1
0.1	Δ*π*_1_	0.00	0.67	1.34	2.01	2.68	3.34	4.01	4.68	5.35	6.02	6.69
	Δ*π*_2_	0.38	−0.29	−0.96	−1.63	−2.30	−2.97	−3.63	−4.30	−4.97	−5.64	−6.31
0.3	Δ*π*_1_	−0.97	−0.43	0.10	0.63	1.16	1.69	2.22	2.75	3.28	3.81	4.34
	Δ*π*_2_	0.61	0.08	−0.45	−0.98	−1.51	−2.04	−2.57	−3.10	−3.63	−4.16	−4.70
0.6	Δ*π*_1_	−2.64	−1.98	−1.33	−0.68	−0.02	0.63	1.28	1.94	2.59	3.24	3.89
	Δ*π*_2_	1.86	1.20	0.55	−0.10	−0.76	−1.41	−2.06	−2.71	−3.37	−4.02	−4.67
0.9	Δ*π*_1_	−3.55	−2.51	−1.48	−0.44	0.59	1.62	2.66	3.69	4.72	5.76	6.79
	Δ*π*_2_	6.17	5.14	4.11	3.07	2.04	1.00	−0.03	−1.06	−2.10	−3.13	−4.16

**Table 6:** Profit increments of two supply chains (*r* = 0.9)

*k*	Profit increments	*λ*										
		0	0.1	0.2	0.3	0.4	0.5	0.6	0.7	0.8	0.9	1
0.5	Δ*π*_1_	−3.69	−2.66	−1.63	−0.59	0.44	1.47	2.51	3.54	4.57	5.61	6.64
	Δ*π*_2_	6.26	5.23	4.20	3.16	2.13	1.10	0.06	−0.97	−2.00	−3.04	−4.07
1	Δ*π*_1_	−3.66	−2.63	−1.60	−0.56	0.47	1.50	2.54	3.57	4.60	5.64	6.67
	Δ*π*_2_	6.25	5.21	4.18	3.15	2.11	1.08	0.05	−0.99	−2.02	−3.05	−4.09
1.5	Δ*π*_1_	−3.61	−2.58	−1.55	−0.51	0.52	1.55	2.59	3.62	4.65	5.69	6.72
	Δ*π*_2_	6.22	5.18	4.15	3.12	2.08	1.05	0.01	−1.02	−2.05	−3.09	−4.12
2	Δ*π*_1_	−3.55	−2.51	−1.48	−0.44	0.59	1.62	2.66	3.69	4.72	5.76	6.79
	Δ*π*_2_	6.17	5.14	4.11	3.07	2.04	1.00	−0.03	−1.06	−2.10	−3.13	−4.16

If *λ* remains unchanged, Δ*π*_1_ will decrease with CI and increase with CGP, and the trend ofΔ*π*_2_ will be the opposite. The weaker the competition, the easier it is for ordinary product chain to get a greater profit improvement from horizontal cooperation, but the green product chain’s profit is seriously damaged. The stronger the competition, the easier it is for two types of products to be substituted for each other, and the more opportunities there will be for two chains to obtain a Pareto improvement in overall profit, as shown in Table 5. But when CI is close to 1, the ability of retailers to obtain a larger share from cooperation becomes a key factor; the more the share, the more profit improvement of the entire chain, but the more the loss of the other chain. Table 6 shows the impact of CGP on profit improvement when CI equals 0.9. The profit improvement of the ordinary product chain has subsequently decreased, while that of the green product chain has increased, but the magnitude of the change is small. CGP does not affect the possibility of Pareto improvement of two chains, but when CI is weak, there is no possibility of a win-win situation for both chains.

## Conclusions and policy implications

This paper focused on horizontal cooperation between retailers in two competing supply chains, one with ordinary products and the other with green ones, to examine how CGP and CI influence decisions like greenness and prices, and related results like demands and profits, and to illustrate whether cooperation can benefit the whole chains and the members, and whether cooperation can be applied as a channel strategy for one of the chains to perform better than the other one. Specifically, the conclusions are as below:

(1) Retail and wholesale prices of green products are always higher than those of ordinary ones. Cooperation will encourage retailers to raise prices and make manufacturers lower wholesale prices. Retail and wholesale prices of green products decrease with CI but increase with CGP, and those of ordinary ones change reversely.

(2) Greenness always increases with CGP but decreases with CI; different from the finding by Murali et al. (2019) that greenness increases with CI. Besides, cooperation is conducive to strengthening the greenness. However, if CI exceeds a certain threshold, the greenness will be weakened by cooperation.

(3) Demand under cooperation, for both types of products, is generally smaller than that without cooperation. Green products’ demand is always larger than ordinary products’ demand. Demand for ordinary products increases with CI but decreases with CGP. Demand for green ones changes with CI convexly but increases with CGP.

(4) Manufacturers’ profits for both types of products under cooperation are always smaller than those under non-cooperation. Green manufacturer’s profit is always higher than ordinary one’s. When retailers cooperate, green manufacturer’s profit increases with CGP and decreases with CI, but ordinary manufacturer’s profit decreases with CGP and changes complexly with multiple inflection points when CI intensifies. Retailers can always benefit from cooperation but manufacturers suffer badly. Similarly, Yang et al. (2017) found that manufacturers’ horizontal cooperation damages retailers’ profit. Differently, they introduced a revenue sharing contract to encourage manufacturers to give up horizontal cooperation. Instead, we proposed that compensating mechanism after retailers’ cooperation may be feasible to achieve a win-win situation for both members in the same chain. Besides, Wu et al. (2018) found that maintaining the same supply chain structure is conducive to coping with horizontal competition and can benefit all participants. The two supply chains in our paper also have the same structure. The difference is that we found that both horizontal cooperation and independent competition have their own advantages for CI is varying.

Specifically, if a retailer selling ordinary products cooperate with a green retailer to sell both types of products, its upstream manufacturer will suffer badly. However, if the retailer can obtain a larger share of profit from cooperation and is willing to give some compensation so that the manufacturer can also benefit from cooperation, the whole chain’s profit will be improved. Based on this logic, in the process of green development, the truth is that manufacturers have to face a quite high cost to implement green strategies. Encouraging retailers to seek cooperation and sell green products together with ordinary ones may be a better way of survival. However, this is not conducive to green manufacturers, and cooperation will seriously damage the profit of green manufacturer and the entire chain. The reason why green refrigerators are not easy to sell may be due to this issue. This is why NIO Company is eager to drive out third-party retailers from the Customer Experience Center, because its new energy vehicles are completely different from ordinary fuel ones, and cooperation will hurt its profits. This is also the reason why Gome deliberately selected tens of retail stores as early as 2012 to promote green products of Haier, Hisense, Samsung, Siemens, and other brands.

A green manufacturer can do the same when CI is strong, encouraging its retailer to cooperate with one ordinary product retailer and try to obtain a larger share from cooperation so that the manufacturer has a chance to be compensated. Cooperation in this situation can bring chances to reach a win-win situation for both chains. This may be the reason why home appliance retailers such as Yongle, Gome, and Suning sell both ordinary products with an energy efficiency tag of level 3 and green ones with a tag of level 2. However, if home appliances with a tag of level 1 are mixed with those with a tag of level 3 or 4, they will only be used as wedding dresses for ordinary products. It is reasonable that Baiguoyuan only sells green fruits, but it sells not only A-level fruits but also AA-level ones. The reason may be that A-level and AA-level fruits are more substitutable, unlike A-level fruits and ordinary ones, the difference between them is not so large, and selling them in the same store increases the possibility that both types of products’ profit can be improved simultaneously.

With respect to the entire competition system, when CI is a little weaker or much stronger, cooperation is helpful to improve the whole system’s profit; but when CI is neither too weak nor too strong, independent operation for each chain is more advantageous; if the state of competition keeps stable and unchanged, independent competition will be also superior to horizontal cooperation between retailers. This provides not only some enlightenment for the competition and cooperation between ordinary and green product chains but also some insights for the development of some industries. For instance, many e-commerce companies initially started from vertical stores, but as the competition became increasingly fierce, they gradually developed into a comprehensive mall, such as JD.com, yhd.com, and Amazon.com. If the state of competition is relatively stable, the living space of vertical stores is relatively large so that they will not be transformed into integrated malls, such as Jumei.com, TrueFacet.com, and womai.com.

### Supplementary Information

ESM 1 (M 2 kb)Chen

ESM 2 (M 32 kb)

ESM 3 (XLSX 77 kb)

## Data Availability

All data generated or analyzed during this study are included in this article and the supplementary files.

## Appendix 1

$$ {\displaystyle \begin{array}{c}A=8\mu +4r-4\mu r+{k}^2r-{k}^2;B=4r+{k}^2r-{k}^2+8;\\ {}C=12\mu r-12r-16\mu -3{k}^2r+6\mu {r}^2-4\mu {r}^3+2{k}^2+4{r}^3+{k}^2{r}^2;D=-2{r}^3+3{r}^2+6r-8;\\ {}\begin{array}{c}E={k}^2{r}^2-3{k}^2r+2{k}^2+4{r}^3+6{r}^2-12r-16;F={k}^2{r}^2-3{k}^2r+2{k}^2+4{r}^3+8{r}^2-8r-16;\\ {}G=8\mu -6\mu r-3\mu {r}^2+2\mu {r}^3+3{r}^2-8;\\ {}\begin{array}{c}H=24\mu +20r-20\mu r+3{k}^2r-3{k}^2-12\mu {r}^2+8\mu {r}^3-8{r}^3+2{k}^2{r}^2-2{k}^2{r}^3;\\ {}I=24\mu -20\mu r+{k}^2r-12\mu {r}^2+8\mu {r}^3+12{r}^2-{k}^2{r}^2-24;\\ {}\begin{array}{c}J=-{r}^3+{r}^2+3r-3;\\ {}K={k}^4{\left(r-1\right)}^2+8{k}^2\mu \left(r-2\right){\left(r-1\right)}^2+24{k}^2r\left(r-1\right)+32\mu \left(1-\mu \right)\left(r-1\right){\left(r-2\right)}^2+80{r}^2+64;\\ {}L=\mu r-2\mu +2;M=2{r}^2+r-4;N=-{r}^2+r+2.\end{array}\end{array}\end{array}\end{array}} $$

## Appendix 2

### Proof of Theorem 1

With respect to Manufacturer 1, the first-order and second-order derivations of profit function on wholesale price is given as follows

$$ \frac{\partial {\pi}_{M1}}{\partial {w}_1}=\frac{\left(2{r}^2-4\right){w}_1+{rw}_2+\left( a\mu {r}^2-{ar}^2-{gkr}^2+ ar-3 a\mu r+3 gkr+2 a\mu -2 gk\right)}{\left({r}^2-4\right)\left(r-1\right)} $$

$$ \frac{\partial^2{\pi}_{M1}}{\partial {w}_1^2}=\frac{2{r}^2-4}{\left({r}^2-4\right)\left(r-1\right)}<0 $$

The second-order derivation is negative, so we can conclude that the profit *π*_*M*1_ has an extreme value.

With respect to Manufacturer 2, the derivations of profit function on wholesale price and greenness are shown as follows

$$ {\displaystyle \begin{array}{c}\frac{\partial {\pi}_{M2}}{\partial {w}_2}=\frac{\left(2{r}^2-4\right){w}_2+r{w}_1+\left( gk{r}^2-3 gkr+2 gk- a\mu {r}^2+3 a\mu r-2 a r-2 a\mu +2a\right)}{\left({r}^2-4\right)\left(r-1\right)}\\ {}\frac{\partial^2{\pi}_{M2}}{\partial {w}_2^2}=\frac{2{r}^2-4}{\left({r}^2-4\right)\left(r-1\right)},\frac{\partial {\pi}_{M2}}{\partial g}=\frac{k{w}_2-4g-2 gr}{r+2},\frac{\partial^2{\pi}_{M2}}{\partial {w}_2\partial g}=\frac{k}{r+2},\frac{\partial^2{\pi}_{M2}}{\partial {g}^2}=-2\end{array}} $$

Next we can get the Hessian matrix below

$$ H=\left|\begin{array}{cc}\frac{\partial^2{\pi}_{M2}}{\partial {w}_2^2}& \frac{\partial^2{\pi}_{M2}}{\partial {w}_2\partial g}\\ {}\frac{\partial^2{\pi}_{M2}}{\partial g\partial {w}_2}& \frac{\partial^2{\pi}_{M2}}{\partial {g}^2}\end{array}\right|=\left|\begin{array}{cc}\frac{2{r}^2-4}{\left({r}^2-4\right)\left(r-1\right)}& \frac{k}{r+2}\\ {}\frac{k}{r+2}& -2\end{array}\right| $$

After simplification, we can obtain

$$ H=-\frac{k^2{r}^2-3{k}^2r+2{k}^2+4{r}^3+8{r}^2-8r-16}{\left(r-1\right)\left(r-2\right){\left(r+2\right)}^2} $$

Specifically, we give the following process

$$ H>0\iff \left({k}^2{r}^2-3{k}^2r+2{k}^2+4{r}^3+8{r}^2-8r-16\right)<0\iff {k}^2<\frac{4\left(r+2\right)\left(2-{r}^2\right)}{\left(1-r\right)\left(2-r\right)} $$

Thus, *π*_*M*2_can be maximized only if$0\le k<2\sqrt{\left(2+r\right)\left(2-{r}^2\right)/\left(2-r\right)\left(1-r\right)}$.

### Proof of Theorem 2

Similarly, we can get the corresponding derivations and matrix as below

$$ {\displaystyle \begin{array}{c}\frac{\partial {\pi}_R}{\partial {p}_1}=\frac{\left(2{p}_1-{w}_1\right)-r\left(2{p}_2-{w}_2\right)}{r-1}+ a\mu - gk,\frac{\partial {\pi}_R}{\partial {p}_2}=\frac{\left(2{p}_2-{w}_2\right)-r\left(2{p}_1-{w}_1\right)}{r-1}-a\left(\mu -1\right)+ gk,\\ {}\frac{\partial^2{\pi}_R}{\partial {p}_1^2}=\frac{2}{r-1}<0,\frac{\partial^2{\pi}_R}{\partial {p}_2^2}=\frac{2}{r-1}<0,\frac{\partial^2{\pi}_R}{\partial {p}_1\partial {p}_2}=-\frac{2r}{r-1}>0,\\ {}H=\mid \begin{array}{cc}\frac{\partial^2{\pi}_R}{\partial {P}_1^2}& \frac{\partial^2{\pi}_R}{\partial {p}_1\partial {p}_2}\\ {}\frac{\partial^2{\pi}_R}{\partial {p}_1\partial {p}_2}& \frac{\partial^2{\pi}_{M2}}{\partial {P}_2^2}\end{array}\mid =\mid \begin{array}{cc}\frac{2}{r-1}& -\frac{2r}{r-1}\\ {}-\frac{2r}{r-1}& \frac{2}{r-1}\end{array}\mid =\frac{4\left(1-{r}^2\right)}{{\left(1-r\right)}^2}>0.\end{array}} $$

Thus, we can conclude that the total profit of retailers is a concave function of retail prices and it can be maximized to get the optimal results.

### Proof of Theorem 3

Definitely, with regard to ordinary manufacturer, we could obtain the derivations of profit function on wholesale price as follows

$$ \frac{\partial {\pi}_{M1}}{\partial {w}_1}=\frac{2{w}_1-{rw}_2- a\mu + a\mu r- gkr}{2\left(r-1\right)} $$, $$ \frac{\partial^2{\pi}_{M1}}{\partial {w}_1^2}=\frac{1}{r-1}<0 $$

They tell us that the profit function can be maximized for it is a concave one. Then we can get the derivations of profit function on wholesale price and greenness and the corresponding matrix for Manufacturer 2 is given as below.

$$ {\displaystyle \begin{array}{c}\frac{\partial {\pi}_{M2}}{\partial {w}_2}=\frac{2{w}_2-r{w}_1+a\left(r-1\right)\left(1-\mu - gk\right)}{2\left(r-1\right)},\frac{\partial {\pi}_{M2}}{\partial g}=\frac{k{w}_2}{2}-2g,\frac{\partial^2{\pi}_{M2}}{\partial {w}_2^2}=\frac{1}{r-1},\frac{\partial^2{\pi}_{M2}}{\partial {g}^2}=-2\\ {}H=\mid \begin{array}{cc}\frac{\partial^2{\pi}_{M2}}{\partial {w}_2^2}& \frac{\partial^2{\pi}_{M2}}{\partial {w}_2\partial g}\\ {}\frac{\partial^2{\pi}_{M2}}{\partial g\partial {w}_2}& \frac{\partial^2{\pi}_{M2}}{\partial {g}^2}\end{array}\mid =\mid \begin{array}{cc}-\frac{1}{r-1}& \frac{k}{2}\\ {}\frac{k}{2}& -2\end{array}\mid =\frac{k^2r-{k}^2+8}{4\left(1-r\right)}\end{array}} $$

It is easy to find that if$0\le k<2\sqrt{2/\left(1-r\right)}$, ∏_*M*2_is a concave function and can be maximized.

### Proof of Proposition 1

According to the preconditions of Theorem 2 and Theorem 3, we can obtain the following results

$$ {\displaystyle \begin{array}{c}\frac{\partial {g}^{\ast }}{\partial k}=\frac{a\left(1-r\right)\left(\mu r-2\mu +2\right)\left(4r-{k}^2r+{k}^2+8\right)}{\left(2-r\right){B}^2}>0,\\ {}\frac{\partial {\pi}_{M1}^{\ast }}{\partial k}=\frac{8{a}^2k{\left(r-1\right)}^2\left(\mu r-2\mu +2\right)\left(4\mu r-4r-{k}^2r-8\mu +{k}^2\right)}{{\left(r-2\right)}^2{B}^2}>0,\\ {}\begin{array}{c}\frac{\partial {\pi}_{M2}^{\ast }}{\partial k}=\frac{2{a}^2k{\left(r-1\right)}^2{\left(\mu r-2\mu +2\right)}^2\left(-4r+{k}^2r-{k}^2+8\right)}{{\left(r-2\right)}^2{B}^2}>0\\ {}\frac{\partial {\pi}_R^{\ast }}{\partial k}=\frac{4{a}^2k{\left(r-1\right)}^2\left(\mu r-2\mu +2\right)\left(-16\mu -4r+8\mu r-{k}^2r+{k}^2+8\right)}{\left(r+1\right){\left(r-2\right)}^2{B}^2}>0.\end{array}\end{array}} $$

### Proof of Proposition 2

$$ \frac{\partial {\pi}_{M2}^{\ast }}{\partial k}=-\frac{2{a}^2k{\left(r-1\right)}^2{\left(\mu r-2\mu +2\right)}^2\left(4r-{k}^2r+{k}^2-8\right)}{{\left(r-2\right)}^2{B}^2} $$

Whether $\frac{\partial {\pi}_{M2}^{\ast }}{\partial k}$is positive depends on whether4*r* − *k*^2^*r* + *k*^2^ − 8is negative.

$$ 4r-{k}^2r+{k}^2-8\ge 0\iff \left(4-{k}^2\right)\left(r-1\right)-4\ge 0\iff {k}^2\ge \frac{4\left(2-r\right)}{1-r} $$

Thus, we can conclude that if$k\ge 2\sqrt{\left(2-r\right)/\left(1-r\right)}$, ${\pi}_{M2}^{\ast }$decrease with*k*. If$0\le k<2\sqrt{\left(2-r\right)/\left(1-r\right)}$,${\pi}_{M2}^{\ast }$increase with*k*. Review Theorem 3, we have $0\le k<2\sqrt{2/\left(1-r\right)}$and find$2\sqrt{\left(2-r\right)/\left(1-r\right)}<2\sqrt{2/\left(1-r\right)}$, then we summarize as

If$0\le k<2\sqrt{2\left(2-r\right)/\left(1-r\right)}$,${\pi}_{M2}^{\ast }$increases with*k*. If $2\sqrt{\left(2-r\right)/\left(1-r\right)}\le k\le 2\sqrt{2/\left(1-r\right)}$, ${\pi}_{M2}^{\ast }$decrease with*k*.

### Proof of Proposition 3.

It is easy to obtain *L* = *μr* + 2(1 − *μ*) > 0, *M* = 2*r*^2^ + *r* − 4 < 0,and*D* = (2 − *r*)*M* < 0.

Moreover, we find

$$ G=-\mu D+3{r}^2-8\le 0\iff \mu \le \left(8-3{r}^2\right)/\left(4-r-2{r}^2\right) $$

$$ B=4r+{k}^2r-{k}^2+8\le 0\iff k\ge 2\sqrt{\left(2+r\right)/\left(1-r\right)} $$

Thus, if *μ* ≤ (8 − 3*r*^2^)/(4 − *r* − 2*r*^2^)and$k\ge 2\sqrt{\left(2+r\right)/\left(1-r\right)}$, there will be *B* ≤ 0, *G* ≤ 0, *LME* > 0, *BG* > 0, *MBE* < 0, refer to the results in Table 4, it is easy to getΔ*g* < 0, Δ*w*_1_ < 0, Δ*w*_2_ < 0, Δ*D*_2_ < 0 **.**

## Footnotes

1 Nike once spent six million US dollars to develop environmentally friendly design tools so that designers can choose fabrics that can reduce environmental impact (http://blog.sina.com.cn/u/1867707644).

2 Marks & Spenser’s environmentally friendly suits are made of Australian organic wool and fabricated in an Italian factory by GOTS dyeing technology. Recycled polyester zippers, recycled linings, buttons and pockets are all completed in a high-tech processing factory in Japan (http://blog.sina.com.cn/u/1867707644).

3 In 2020, quite a few air-conditioning manufacturers cooperated with Gome to carry out green development, for example, Gome grandly launched a Green Consumption Week with an old-for-green campaign in early July (https://www.163.com/tech/article/FFNMT64400099A7M.html).

4 30 companies such as Brilliance Automobile, Guangzhou Honda, and PSA Peugeot Citroen had obtained the production qualification of new energy vehicles, but had not produced and sold new energy vehicles for 1 year or more so that they were criticized by the Ministry of Industry and Information Technology of China in 2017 (https://www.miit.gov.cn).

5 With respect to household appliances such as refrigerators, washing machines, water heaters, and range hoods, China has its own energy consumption standards, which are denoted as 5 different energy efficiency levels, of which level 3 is the average level of energy consumption, marked as yellow; both level 4 and level 5 are worse than the average level and are marked in orange and red; level 2 surpasses the average level and belongs to the domestic advanced level, marked as light green; level 1 means reaching the international advanced level, marked as heavy green.
